# The coherence between PSMC6 and α-ring in the 26S proteasome is associated with Alzheimer’s disease

**DOI:** 10.3389/fnmol.2023.1330853

**Published:** 2024-01-31

**Authors:** Jing Xiong, Xinping Pang, Xianghu Song, Lin Yang, Chaoyang Pang

**Affiliations:** ^1^College of Computer Science, Sichuan Normal University, Chengdu, China; ^2^West China School of Basic Medical Sciences and Forensic Medicine, Sichuan University, Chengdu, China

**Keywords:** Alzheimer’s disease, proteasome, support vector machine, entropy, determinant

## Abstract

Alzheimer’s disease (AD) is a heterogeneous age-dependent neurodegenerative disorder. Its hallmarks involve abnormal proteostasis, which triggers proteotoxicity and induces neuronal dysfunction. The 26S proteasome is an ATP-dependent proteolytic nanomachine of the ubiquitin-proteasome system (UPS) and contributes to eliminating these abnormal proteins. This study focused on the relationship between proteasome and AD, the hub genes of proteasome, PSMC6, and 7 genes of α-ring, are selected as targets to study. The following three characteristics were observed: 1. The total number of proteasomes decreased with AD progression because the proteotoxicity damaged the expression of proteasome proteins, as evidenced by the downregulation of hub genes. 2. The existing proteasomes exhibit increased activity and efficiency to counterbalance the decline in total proteasome numbers, as evidenced by enhanced global coordination and reduced systemic disorder of proteasomal subunits as AD advances. 3. The synergy of PSMC6 and α-ring subunits is associated with AD. Synergistic downregulation of PSMC6 and α-ring subunits reflects a high probability of AD risk. Regarding the above discovery, the following hypothesis is proposed: The aggregation of pathogenic proteins intensifies with AD progression, then proteasome becomes more active and facilitates the UPS selectively targets the degradation of abnormal proteins to maintain CNS proteostasis. In this paper, bioinformatics and support vector machine learning methods are applied and combined with multivariate statistical analysis of microarray data. Additionally, the concept of entropy was used to detect the disorder of proteasome system, it was discovered that entropy is down-regulated continually with AD progression against system chaos caused by AD. Another conception of the matrix determinant was used to detect the global coordination of proteasome, it was discovered that the coordination is enhanced to maintain the efficiency of degradation. The features of entropy and determinant suggest that active proteasomes resist the attack caused by AD like defenders, on the one hand, to protect themselves (entropy reduces), and on the other hand, to fight the enemy (determinant reduces). It is noted that these are results from biocomputing and need to be supported by further biological experiments.

## 1 Introduction

As the most prominent form of dementia, Alzheimer’s disease (AD) is a chronic neurodegenerative disorder seen in age-dependent (De-Paula et al., 2012; Guo et al., 2020; Zhang et al., 2022a; Ashleigh et al., 2023). Typical clinical manifestations of AD characterized by progressive impairment of episodic memory and cognitive function, eventually evolve into disruption of fundamental functions (Long and Holtzman, 2019; Silva et al., 2019; Penney et al., 2020). Since the pathobiology of the disease is heterogeneous, clear pathogenesis remains elusive (Scheltens et al., 2021; Zhang et al., 2022b; Wenbo, 2023; Yang L. et al., 2023).

Anatomically, the insoluble amyloid-beta peptides (Aβ) in extracellular neuritic plaques and the hyperphosphorylated microtubule-associated Tau protein in intraneuronal neurofibrillary tangles (NFT) are the two neuropathological hallmarks in AD (Busche and Hyman, 2020; Yang X. et al., 2023). At the molecular level, this pathological process begins with the self-assembly of abnormal soluble oligomers (Cataldi et al., 2021). When the rate of aggregation of these misfolded proteins exceeds the rate of metabolic transformation in the organism, intracellular protein homeostasis is damaged. This causes an accumulative toxicity effect, thereby forming toxic substances and causing neurocytotoxicity (Ait-Bouziad et al., 2017; Lee et al., 2020; Liang et al., 2022). Multiple studies have corroborated a clear association of neurotoxicants with AD pathogenicity (Nisa et al., 2021). They induce the dysfunction of many cellular processes involved in the pathogenesis of AD (Schmidt et al., 2021). Thus, Inhibiting the toxicity of these oligomers is an effective strategy to slow disease progression (Tolar et al., 2021; Colom-Cadena et al., 2023).

These abnormal proteins are usually characterized by ubiquitin positivity (Callis, 2014). A major purpose of ubiquitin signaling is to target aberrant proteins to the clearance system for degradation (Le Guerroué and Youle, 2021). The human clearance system mainly consists of the ubiquitin (Ub)-proteasome system (UPS) and the autophagy-lysosomal pathway (ALP). They maintain protein quality control. Among them, the UPS is a highly sophisticated supramolecular complex shaped like a barrel containment (Snyder et al., 2005). It is responsible for degrading short-lived, damaged, and misfolded proteins located in the nucleus and cytoplasm (Livneh et al., 2016; Murata et al., 2018). And that most cellular proteins in eukaryotes target the ATP-dependent 26S proteasome (Finley, 2009; Zhang et al., 2022c). Numerous studies have shown that the proteasome is implicated in neuroplasticity and neurodegeneration (Küry et al., 2017; Fernández-Cruz and Reynaud, 2021). Because it can be recruited to the synapse to regulate the localized turnover of pre- and post-synaptic proteins (Livneh et al., 2016). In addition, cytotoxic tau proteins in AD inclusion bodies are particularly sensitive to proteolytic by the ubiquitin-proteasome (Babu et al., 2005; Tai et al., 2012; Myeku et al., 2016; Ukmar-Godec et al., 2020; Horie et al., 2021) system. Experiments have proved that enhancing proteasome can improve AD-like pathology (Chocron et al., 2022). At the same time, the proteasome can be affected by abnormal proteins. On the one hand, aberrant proteins can directly allosteric 20S and inhibit 20S proteasome (Thibaudeau et al., 2018). On the other hand, toxicity causes synaptic degeneration (Tzioras et al., 2023), indirectly impedes interneuronal proteasome communication and transport, disrupts protein homeostasis, and leads to the disintegration of the ubiquitin-proteasome system (Liu et al., 2019). Therefore, it would be interesting to explore how the over-accumulation of toxicants in the AD process damages the proteasome and what the proteasome does in response to maintain homeostatic function.

This paper is dedicated to identifying the functional mechanisms of the proteasome in relationship to the AD process. PSMC6 and the α-ring (PSMA1-PSMA7) were used as the research targets. In this study, we observed that the decreased expression and increased correlation of proteasomal subunits were strongly associated with AD. Therefore, we tried to investigate the effect of over-accumulated toxic proteins on the 26S proteasome and the feedback of the proteasome on proteostasis in the AD process. It is hoped to provide a valuable reference for AD diagnosis.

## 2 Materials and methods

**FIGURE 1:**
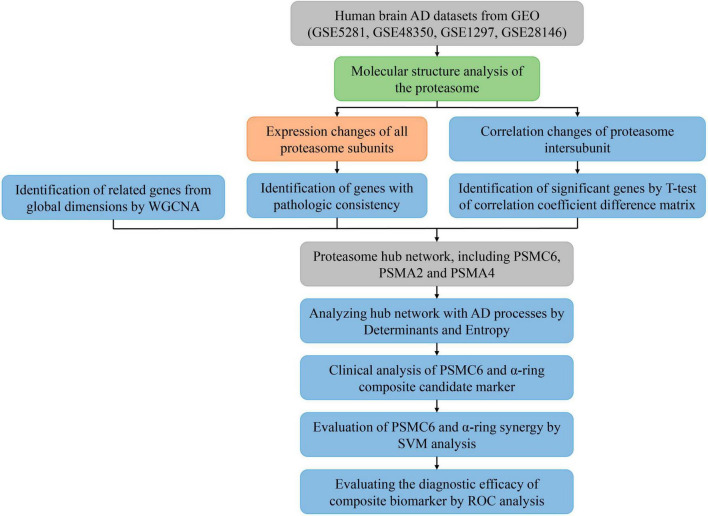
Flowchart of the analysis process. Different colors represent different types of analysis. Where input and output data are represented in gray, descriptive analysis is shown in light green, comparative analysis to validate known results is represented in orange, and the blue modules are used to represent the search for unknown results, indicating exploratory analysis.

The flowchart of the bioinformatics analyses combined with machine learning and innovation strategies is shown in Figure 1.

### 2.1 Data sources and organization

The relevant genes used in this study were collected from the National Center for Biotechnology Information (NCBI) database. The Gene Expression Omnibus (GEO) public database^1^ was used to download human gene expression profiles GSE5281, GSE48350, GSE28146, and GSE1297 data. GSE5281, GSE28146, and GSE48350 are brain tissue section data constructed on the Affymetrix Microarrays GPL570 platform. The dataset GSE5281 covers six brain tissue regions. It includes 74 control samples and 87 samples from AD patients. GSE48350 collected 253 samples from four different regions, including 80 samples from the AD group. GSE28146 contains 30 samples from the hippocampus containing 8 normal and 22 with different disease severity. GSE1297 was constructed based on the GPL96 platform. It analyses gene expression in the hippocampus of 9 control subjects and 22 subjects with AD of varying severity on 31 separate microarrays. This dataset also includes clinical information such as the Minimal State Examination (MMSE) and neurofibrillary tangles (NFT) and other clinical information. For detailed information, refer to Table 1. For a group with a given sample size *n*, if the number of genes is *m*, it can be represented by a gene expression matrix *G* that:


$$G={\left(g_{i\,j}\right)}_{m\times n}$$

where *g*_*ij*_ denotes the expression of the *i*th gene in the *j*th sample, namely, each row in the matrix represents a gene and each column represents a sample.

**TABLE 1:** Detailed characteristics of the datasets.

Dataset	Platform	Tissue	Clinical indicators	Platform	Samples
GSE5281	GPL570	6 brain regions	No	GPL570	161
GSE48350	GPL570	4 brain regions	No	GPL570	253
GSE28146	GPL570	CA1 hippocampal gray	No	GPL570	30
GSE1297	GPL96	Hippocampus	MMSE and NFT scores	GPL96	31

### 2.2 Data pre-processing

Given the different conditions of the microarray experiments, the expression range of the data varied greatly. Before statistical analysis, all gene expression profiles were logarithmically processed with a base of 2 (denoted by the matrix *X*). Then, a normalized output (notated as the matrix *Z*) was made for each sample sequence in the GSE1297 dataset using the Z-score. For expression profiles GSE5281 and GSE48350, the expression intensities were normalized using the “normalizeBetweenArrays” function in the “limma” package of the R (version 4.2.1) software (denoted by the matrix *Q*). Final filtering and annotation of gene expression profiles.

### 2.3 Statistical analysis

The proteasome expression pattern in the AD process was determined by analyzing the differences in 41 proteasomal assembly subunits between the case (*Q*_*AD*_) and control (*Q*_*CON*_) groups in GSE5281 and GSE48350. To further identify hub genes of the proteasome, Pearson’s Difference Correlation Coefficient Matrix was analyzed for each group of the GSE1297 dataset with the *T*-test.

#### 2.3.1 *T*-test

*T*-test, also called Student’s *t*-test, the premise is that the sample is required to follow a normal distribution or near normal distribution. According to the research design, the One-sample *t*-test was chosen, based on the difference between the sample mean and the overall mean to construct the t-statistic to assess the significance of the difference. The algorithm flow for one-sample *t*-test is shown as Algorithm 2-1:

**Algorithm 2-1:** One-sample *T*-test.

Inputs: The set of observed samples Φ_*n*_, the set of aggregate data Φ_*m*_, and the significance level α, where *n* and *m* are the number of samples Outputs: Acceptance or rejection of the original hypothesis Steps: 1 Calculate the sample mean and the overall mean: $\bar{x}=\frac{1}{n}\,\sum_{i=1}^{n}x_{i}$, $\mu =\frac{1}{m}\,\sum_{i=1}^{m}x_{i}$ 2 Calculate the variance of the samples: $s=\sqrt{\frac{1}{n-1}\,\sum_{i=1}^{n}{\left(x_{i}-\bar{x}\right)}^{2}}$ 3 Construct the t-statistics: $t=\frac{\bar{x}-\mu }{s/\sqrt{n}}$ 4 Locate the critical value and calculate the *p*-value according to the degrees of freedom *df* = *n*−1 5 Comparison of *p*-value and significance level α

Given significance level α, also known as the probability of Type I error. In this study, the predetermined significance level α = 0.05.

#### 2.3.2 Pearson correlation coefficient matrix

For the sets of correlation coefficient matrices, we extracted the raw matrices of the four stages of the 41 proteasomal subunits as inputs, denoted as *G*_*CON*_, *G*_*INC*_, *G*_*MOD*_ and *G*_*SEV*_. The algorithm for Pearson’s correlation coefficient matrix is as follows:

**Algorithm 2-2:** Pearson correlation coefficient matrix.

Inputs: Gene expression matrix *G*_*m × n*_, where each row *i* represents a gene and each column *j* represents a sample Outputs: Gene correlation coefficient matrix *R*_*m × m*_ Steps: 1 Calculate the mean ${\bar{X}}_{i}$ and overall standard deviation σ_*i*_ for each row (each gene) across all samples: for each gene *i*, ${\bar{X}}_{i}=\frac{1}{n}\,\sum_{j=1}^{n}G_{i\,j}$, $\sigma_{i}=\sqrt{\frac{1}{n}\,\sum_{j=1}^{n}{\left(G_{i\,j}-{\bar{X}}_{i}\right)}^{2}}$ 2 Iterate through any two rows (gene *i* and gene *j*), calculate the covariance: $c\,o\,v_{i\,j}=\frac{1}{n}\,\sum_{k=1}^{n}\left(G_{i\,k}-{\bar{X}}_{i}\right)\,\left(G_{j\,k}-{\bar{X}}_{j}\right)$ 3 Calculate the overall correlation coefficient for gene *i* and gene *j*: $\rho_{i\,j}=\frac{c\,o\,v_{i\,j}}{\sigma_{i}\,\sigma_{j}}$ 4 Construct the final Pearson correlation coefficient matrix *R* = (ρ_*ij*_)_*m*×*m*_

The four correlation coefficient matrices, denoted *R*_*CON*_, *R*_*INC*_, *R*_*MOD*_ and *R*_*SEV*_, were derived separately from Algorithm 2-2. All four matrices are 41×41 symmetric square matrices, and each element represents the linear correlation coefficient of two proteasome subunits. Given the symmetry of the correlation coefficient matrix, the statistical histograms of the upper triangular elements of the correlation coefficient matrices ($R_{\mathrm{CON}}^{u}$, $R_{\mathrm{INC}}^{u}$, $R_{\mathrm{MOD}}^{u}$, and $R_{\mathrm{SEV}}^{u}$) for each period were plotted separately by using the SPSS software (version 26.0.0.0) and observed the variability of the disease periods.

#### 2.3.3 *T*-test of the correlation coefficient difference matrix

The formula for the difference matrix is as follows:


$$△\,R=R_{s\,t\,a\,g\,e}-R_{C\,O\,N},s\,t\,a\,g\,e=I\,N\,C,M\,O\,D,S\,E\,V$$

The final set of differences obtained are denoted as △*R*_*INC*−*CON*_, △*R*_*INC*−*CON*_, and △*R*_*INC*−*CON*_, respectively. And the statistical histograms of the respective upper triangular matrix elements are again described.

It is further found that the histogram matches the characteristics of the T-distribution with a probability density function of:


$$f\,\left(t\right)=\frac{\Gamma \,\left(\left(v+1\right)\right)/2}{\sqrt{v\,\pi }\,\Gamma \,\left(v/2\right)}\,{\left(1+t^{2}/v\right)}^{-\left(v+1\right)/2}$$

where *v* is called the degree of freedom and Γ is the gamma function. The *t*-value corresponds to the value of the horizontal coordinate, and assuming that the *t*-value is *a*, then the area under the curve after *t* = *a* is actually the *p*-value. The larger the degree of freedom, the closer *f*(*t*) is to the standard normal distribution. The normal distribution is:


$$X∼N\,\left(\mu ,\sigma^{2}\right)$$

where *u* is the mean and σ is the standard deviation. In a normal distribution, the probability that a value is distributed in (μ−σ,μ + σ) is 0.6826; the probability that a value is distributed in (μ−2σ,μ + 2σ) is 0.9544; and in (μ−3σ,μ + 3σ) is 0.9974. The 3σ principle can be simply described as follows: if the data follow a normal distribution, an outlier is defined as a value in a set of resultant values that deviates from the mean by more than three times the standard deviation. The principle is specified as follows:


P(|x-μ|>σ)≤0.318

P(|x-μ|>2σ)≤0.046

P(|x-μ|>3σ)≤0.003

values exceeding 2 times the standard deviation screened according to 3σ were considered significant (*p* < 0.05). Therefore, for each of the three sets (△*R*_*INC*−*CON*_, △*R*_*MOD*−*CON*_, and △*R*_*SEV*−*CON*_), a subset of each set beyond 2σ was extracted and denoted as Δ*S*_*INC*−*CON*_, Δ*S*_*MOD*−*CON*_, and Δ*S*_*SEV*−*CON*_. Take the intersection of these three sets:


$$\Omega =\Delta \,S_{I\,N\,C-C\,O\,N}\,⋂\Delta \,S_{M\,O\,D-C\,O\,N}\,⋂\Delta \,S_{S\,E\,V-C\,O\,N}$$

four proteasome hyperactive genes were eventually available: PSMA2, PSMA4, PSMC6, and PSME1.

### 2.4 The system determinant

The determinant is an important linear algebra concept that has a wide range of applications in both mathematics and engineering. At the level of linear algebra, if the determinant of a matrix is zero, it means that the matrix is singular and its column vectors are linearly related. From a vector spaces perspective, if the rows (or columns) of a matrix are the basis of a vector space, then the value of the determinant indicates the volume or extent of the vector space into which these vectors are ten sored. The plus or minus sign indicates direction.

**FIGURE 2:**
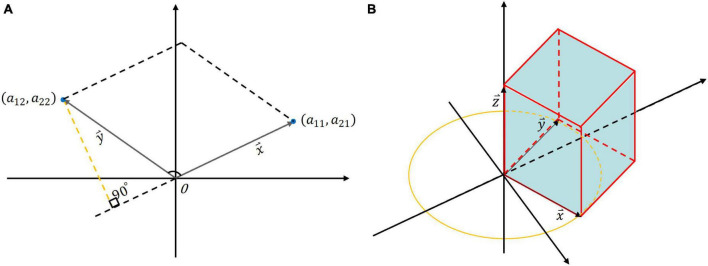
The geometric interpretation of determinant: **(A)** The area of the quadrilateral formed by the two column vectors of a 2 × 2 matrix; **(B)** The volume of a 3 × 3 matrix where column vectors are orthogonal and lie on coordinate axes.

Specifically, suppose a second-order matrix *A* consisting of $\overset{⇀}{x}$ and $\overset{⇀}{y}$ column vectors:


$$A=\left[\begin{array}{cc} a_{11}\; & a_{12}\\ a_{21}\; & a_{22} \end{array}\right]$$

as shown in Figure 2A. We designate one side as the base and draw a perpendicular line from another side to this base. The formula for calculating the area of the parallelogram is as follows:


$$S_{A}=\left|\vec{x}\right|\,\left|\vec{y}\right|\,s\,i\,n\,\theta =\left|\vec{x}\right|\,\left|\vec{y}\right|\,\sqrt{1-{\left(\frac{\vec{x}\cdot \vec{y}}{\left|\vec{x}\right|\,\left|\vec{y}\right|}\right)}^{2}}=\left|a_{11}\,a_{22}-a_{12}\,a_{21}\right|$$

hence, the area of the parallelogram formed by the column vectors of *A* is precisely equal to the determinant of *A*. If the determinant of a 2 × 2 matrix is smaller, it signifies that the angle between the two column vectors is smaller, indicating a stronger correlation between the two vectors. Subsequently, extending the derivation to a 3 × 3 matrix *B*:


$$B=\left[\begin{array}{ccc} a_{11}\; & a_{12}\; & a_{13}\\ a_{21}\; & a_{22}\; & a_{23}\\ a_{31}\; & a_{32}\; & a_{33} \end{array}\right]=\left[\begin{array}{ccc} \vec{x}\; & \vec{y}\; & \vec{z} \end{array}\right]$$

if the column vectors in matrix *B* are orthogonal and each column vector lies along a coordinate axis, in this particular scenario, the volume enclosed by the column vectors forms a rectangular parallelepiped, the volume of which is equivalent to the scalar triple product of the three column vectors (Figure 2B):


$$V_{B}=\vec{x}\cdot \left(\vec{y}\times \vec{z}\right)$$

When one vector, says $\overset{⇀}{z}$, projects onto the plane formed by the other two vectors ($\overset{⇀}{x}$ and $\overset{⇀}{y}$) with a longer projection or. In other words, a smaller angle between the vector and the plane indicates a higher degree of correlation among the three vectors. So, the value of the determinant represents the area of a parallelogram (in the two-dimensional case) or the volume of a parallel hexahedron (in the three-dimensional case). The notion of determinant can also be generalized to the higher-dimensional case, representing the hypervolume into which these vectors are tensioned.

The Pearson correlation coefficient matrices for each stage of the significant gene networks PSMA2, PSMA4, and PSMC6 were analyzed to extract valuable information for the determinant of the network system at each stage. For instance, for the normal phase, the matrix of correlation coefficients for the hub gene network (PSMA2, PSMA4, PSMC6) is denoted as $R_{C\,O\,N}^{h\,u\,b}$. Then the $d\,e\,t\,\left(R_{C\,O\,N}^{h\,u\,b}\right)$ is the volume of the parallel hexahedron enclosed by the three-dimensional column vectors of PSMA2, PSMA4, and PSMC6.

### 2.5 Entropy

Entropy involves a wide range of fields such as information theory, thermodynamics, statistical physics, information science, and ecology. It explains uncertainty, chaos, and diversity from different perspectives. Specifically, in information theory, entropy indicates the uncertainty or the amount of information in a random variable. The greater the entropy, the more uncertain the random variable, and vice versa. In thermodynamics, entropy is a state function of a system that is used to describe the degree of chaos or disorder of the system. The more disordered the system, the greater its entropy value. In statistical physics, entropy is related to the number of microstates, and the principle of entropy increase states that entropy does not decrease in isolated systems. In ecology, entropy is often used to describe the diversity and stability of ecosystems. A low entropy system has higher stability and adaptability. In information theory, normalized mutual information is first calculated as follows. Let *X* be a discrete random variable with the following distribution function:


$$\begin{array}{ccc} X & x_{1} & x_{2}\,\cdots \,x_{n}\\ Q & p_{1} & p_{2}\,\cdots \,p_{n} \end{array}$$

*X* is an assessment parameter, *n* is the number of categories for that assessment parameter, and *p_i_* denotes the frequency of category *x_i_*. The entropy of the random variable *X* is determined by the following equation:


$$H\,\left(X\right)=-\sum_{i=1}^{n}P\,\left(x_{i}\right)\,l\,o\,g_{2}\,P\,\left(x_{i}\right)$$

The spectral theorem for real symmetric matrices states that the eigenvalues are necessarily real numbers. Therefore, the system entropy of the hub gene network is calculated as shown in Algorithm 2-3:

**Algorithm 2-3:** System entropy.

Inputs: Correlation coefficient matrix for hub gene networks *R*_*n × n*_ Outputs: The entropy *H*(*P*) of the matrix *R* Steps: 1 Eigenvalue decomposition of *R*: *R* = *Q*Λ*Q*^−1^, where Λ is a diagonal matrix whose elements on the diagonal λ are the eigenvalues of *R*, and *Q* is an eigenvector matrix with each column corresponding to an eigenvector, then obtain ordered eigenvalues λ_1_ ≥ λ_2_ ≥ ⋯ ≥ λ_*n*_ 2 Get the eigenvalue percent occupancy vector *P* = {*p*_1_,*p*_2_,…,*p*_*n*_}: $P_{i}=\frac{\sqrt{\lambda_{i}^{2}}}{\sqrt{\sum_{i=1}^{n}\lambda_{i}^{2}}}$ 3 Calculating discrete forms of system entropy: $\text{H}\,\left(P\right)=-\sum_{i=1}^{n}P\,\left(x_{i}\right)\,\log_{2}P\,\left(x_{i}\right)$

**FIGURE 3:**
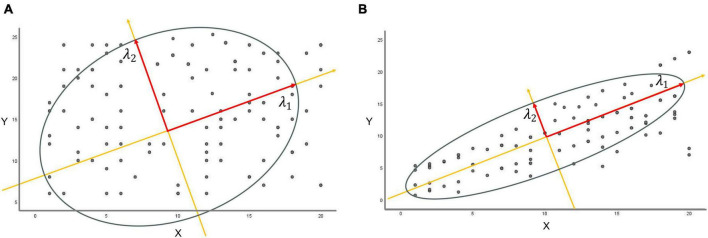
The relationship between eigenvalues and entropy. **(A)** Constructing a new coordinate system from the first two eigenvalues, λ_1_ and λ_2_, if the difference between λ_1_ and λ_2_ is relatively small, the data points are uniformly distributed in both directions of the new coordinates without evident major or minor axes. **(B)** When the eigenvalue λ_1_ greatly exceeds eigenvalue λ_2_, the matrix predominantly exhibits significance or dominance in the principal direction of major variation (long axis λ_1_), leading to substantial variations in data along this particular direction.

In terms of statistics, the sampled distribution of data points exhibits a certain degree of correlation, aligning successively along distinct feature axes. λ_1_ represents the longest principal axis, λ_2_ denotes the secondary semiaxis orthogonal to it within the same plane, and λ_3_ constitutes the third orthogonal feature axis perpendicular to this plane, and so forth. When entropy increases, the principal eigenvalues lack significant differences. This emphasizes the independence of the range of data point distributions. Conversely, as the eigenvalue λ_1_ grows larger, the matrix exhibits pronounced dominance or significance in the primary direction of variation. This results in a denser concentration of data points near the principal axis, indicating a more organized data arrangement. Correspondingly, the entropy diminishes (Figures 3A, B).

For the information entropy of the determinant, enumerated all combinations of the specified dimension (number of samples, denoted as dim, *dim* = 3,4,5). For a matrix with *n* samples, there are a total of $C_{n}^{d\,i\,m}$ combinations, and then the matrix of correlation coefficients for each combination is computed using algorithm 2-2. This resulted in four groups of determinant sets, denoted $\Omega \,\left(d\,e\,t\,\left(R_{c\,o\,n}^{h\,u\,b}\right)\right)$, $\Omega \,\left(d\,e\,t\,\left(R_{i\,n\,c}^{h\,u\,b}\right)\right)$, $\Omega \,\left(d\,e\,t\,\left(R_{m\,o\,d}^{h\,u\,b}\right)\right)$, and $\Omega \,\left(d\,e\,t\,\left(R_{s\,e\,v}^{h\,u\,b}\right)\right)$. Then, we plotted a statistical histogram for each group of determinant sets based on the minimum value, maximum value, and the partition interval constant *a* for each group. To ensure objectivity, we set the interval to:


$$a=\sigma \,\left(\Omega \,\left(d\,e\,t\,\left(R_{c\,o\,n}^{h\,u\,b}\right)\right)⊕\Omega \,\left(d\,e\,t\,\left(R_{i\,n\,c}^{h\,u\,b}\right)\right)⊕\Omega \,\left(d\,e\,t\,\left(R_{m\,o\,d}^{h\,u\,b}\right)\right)⊕\Omega \,\left(d\,e\,t\,\left(R_{s\,e\,v}^{h\,u\,b}\right)\right)\right)/10$$

where ⊕ denotes the union of two sets and the same elements can be repeated. σ is the standard deviation, taken as one-tenth of the standard deviation of the multiset.

For each group, excluding intervals with a frequency of 0, each interval is set to a random variable *x_i_*, the frequency corresponding to the valid interval is the probability of the random variable *p_i_*. The Shannon entropy was then used to quantify the orderliness of the groups. We observed that both the entropy of the system and the entropy of the determinant decreased consistently with the development of the disease, and the more samples were taken, the more pronounced the characteristics were.

### 2.6 Weighted gene co-expression network analysis

We employed weighted gene co-expression network analysis (WGCNA) to elucidate important gene expression modules. Using the dataset GSE1297, the absolute median MAD of each gene was first calculated separately. Then we excluded the top 50% of genes with the smallest MAD. The outliers and samples were removed by utilizing the “goodSamplesGenes” function. Then the minimum number of genes in a module was set to 20, and finally, a collection of genes with 16 co-expression modules was obtained to analyze the relationship between each module and the clinical features MMSE, NFT, and Braak and visualize the clustering by the Sangerbox platform^2^ to visualize the clustering analysis results (Shen et al., 2022).

### 2.7 Principal component analysis

Principal component analysis (PCA) can reduce the feature dimension of high-dimensional data while retaining the main information of the data. All logarithmized sample data sets consisting of the α-ring (PSMA1-PSMA7) and PSMC6 in GSE1297 were analyzed by principal component analysis using the “prcomp” function of R software, and after obtaining the transformed new coordinate system. The first principal component with the largest eigenvalue was taken to be the first principal component of the PC1 vector.

### 2.8 Support vector machine

The support vector machine (SVM) is a machine learning algorithm for binary and multi-classification problems. In this study, we used the svm.SVC class from the Scikit-learn library in Python (version 3.8.5) to construct an SVM classifier with a linear kernel by setting the parameter kernel = “linear.” We used the GSE5281 dataset with the expression of PSMC6 and the mean α ring as inputs to distinguish normal cases from AD. To evaluate the performance of the classifier, the StratifiedKFold ten-fold cross-validation method was used to assess the classifier performance. The tools for model quality and performance evaluation are the Receiver Operating Characteristic Curve (ROC) and the Area Under the Curve (AUC). The experimental results show that our constructed SVM classifier showed good performance in this task.

## 3 Results

### 3.1 The structure of the proteasome

**FIGURE 4:**
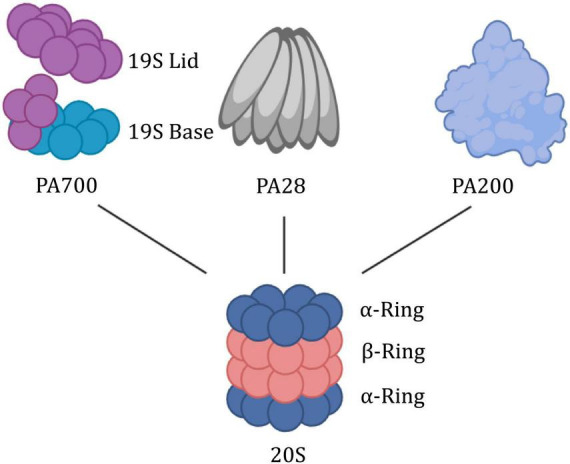
Building block concept of the proteasome system. The 20S proteasome is symmetrically built of two outer rings consisting of alpha subunits and two inner rings built from beta subunits. Via its α-ring surface, it binds proteasome activators such as the PA700, PA28αβγ, or PA200. PA700, also termed 19S RP, has a lid region that recognizes and binds to polyubiquitin substrate proteins, thereby allowing the substrate proteins to unfold and be transported through the base region with ATPase activity.

As a kind of garbage collector within the cell, the proteasome is tightly regulated by numerous subunits, which share a common proteolytic core, the 20S proteasome (Figure 4). The 20S is composed of a cylindrical axially stacked of four hetero-oligomeric rings (Li et al., 2015). The outer α-ring contains seven similar, yet distinct α-subunits (α1-α7). It forms a tightly regulated entrance gate of substrates. Similarly, the inner constitutive β-ring consists of seven distinct β-subunits (β1-β7). The β-ring contains 6 hydrolysis sites to shear incoming substrates. There are other subunits (PSMA8, PSMB8-PSMB10) that are expressed as tissue-specific (Wang et al., 2020). It showed that elevated 20S proteasome levels facilitate survival under proteotoxic stress (Sahu et al., 2021). And its activation promotes longevity extension and resistance to proteotoxicity in Caenorhabditis elegans (Chondrogianni et al., 2015).

The mechanism of the CP-gate opening and proteasome activity is regulated by the attached docking station (19S RP, PA28αβ, PA28γ, and PA200). 19S, as a canonical proteasome-activated cap, is further divided into two additional subcomplexes, the “base” and “lid.” The base consists of heterohexameric motor AAA ATPase particles (PSMC1-PSMC6) and four regulatory particle non-ATPase subunits (PSMD1, PSMD2, and PSMD4). Among them, six AAA ATPase particles are organized into a spiral-stepped RPT ring that delivers high-energy nucleotides (Livneh et al., 2016). The lid which serves to recognize and remove ubiquitin, is composed of nine different PSMD subunits (PSMD3, PSMD6-8, PSMD11-15) in a horseshoe-shaped structure. Particularly, PSMD5, PSMD9, PSMD10, and PSMD14 serve as activating assembly factors. Tsvetkov noted that inhibition of the 19S regulatory complex increases cell survival when the proteasome is inhibited to toxic levels (Tsvetkov et al., 2015). The PA28αβγ activator is formed by seven alpha (PSME1), beta (PSME2), or gamma (PSME3) subunits, respectively, or a mixture of both, while PA200 (PSME4) is a highly conserved monomeric activator (Wang et al., 2020).

The protein encoded by PSMC6 is one of the 19S RPT subunits of the proteasome, and the yeast homolog is RPT4. Structurally, the RPT ring anchors the 19S to the 20S to form the 26S proteasome. It is the intermediary bridge between the substrate from recognition to entry into the hydrolysis chamber (Dong et al., 2019; Zhang et al., 2022c). More strategically, as a proteasomal AAA+ ATPase molecular motor, the RPT ring releases energy and exerts mechanical tension through ATP hydrolysis. This process is used to stimulate the activity of deubiquitinase (DUB) Rpn11 (Lipson et al., 2008; Matyskiela et al., 2013), drives the unfolding and translocation of substrate protein (Bard et al., 2018; Dong et al., 2019), and direct its terminal conformational changes to open the α-ring gated channel (Smith et al., 2007; Lasker et al., 2012; Finley et al., 2016). As the only RPT subunit without CP insertion (Zhu et al., 2018), PSMC6 may be a pivotal anchor for the flexible bolstering of the two interfaces in the highly dynamic mechanism of 26S. Recent studies have demonstrated that PSMC6 overexpression could impair cell cycle progression and cell proliferation. However, the Silence of PSMC6 Inhibits Cell Growth and Metastasis in Lung Adenocarcinoma (Zhang et al., 2021). α-ring is composed of the PSMA family, which is the first step of 20S assembly and provides a structural template, but the whole assembly process is still unclear (Schnell et al., 2021, 2022). Numerous studies have been conducted on PSMA as bait proteins of neurotoxic ligands of ataxic proteins (Lim et al., 2006; Hosp et al., 2015; Ulbrich et al., 2018).

### 3.2 The downregulation characteristic of proteasome subunits in AD

**FIGURE 5:**
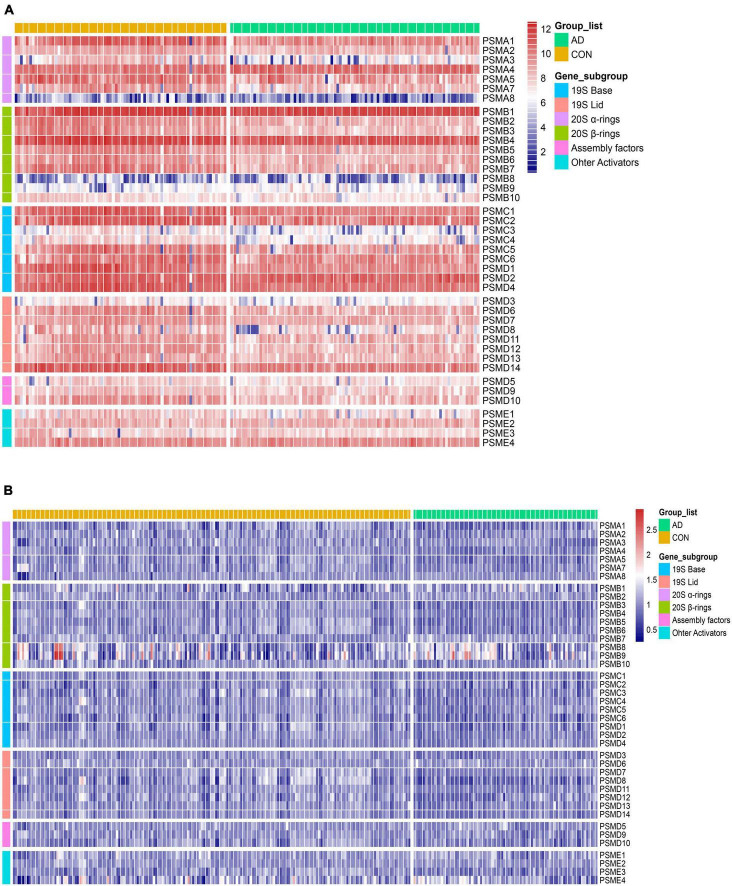
Heat map of differential expression of proteasome subunits. **(A,B)** From left to right, heatmaps of the differential expression of 41 proteasome subunits in the datasets GSE5281 and GSE48350. Respectively, arranged from top to bottom by gene order of α-ring, β-ring, base, lid, assembly factors, and other activators. *Compared to the CON, the expression of the AD group was generally downregulated*.

To measure aberrations of the proteasome in AD, the expression of all proteasome subunits in two different datasets (GSE48350 and GSE5281) was visualized using heat maps. Then, two independent sets of expression heat maps were obtained (Figures 5A, B). The majority of proteasome subunits were down-expression in the AD patient group compared to normal brains. As AD progresses, abnormal proteins accumulate, and excessive accumulation leads to proteotoxicity. The Proteotoxicity results in damage to the proteasome, which leads to aberrant expression of proteasomal subunits. As a result, the subunits hold the characteristic of significant downregulation. That is, the total number of proteasomes is insufficient to degrade abnormal proteins. This reduction in degradation leads to over-accumulation of abnormal proteins and damages cellular proteostasis. Thus, AD exacerbates.

### 3.3 Increased proteasome intersubunit correlation in AD

**FIGURE 6:**
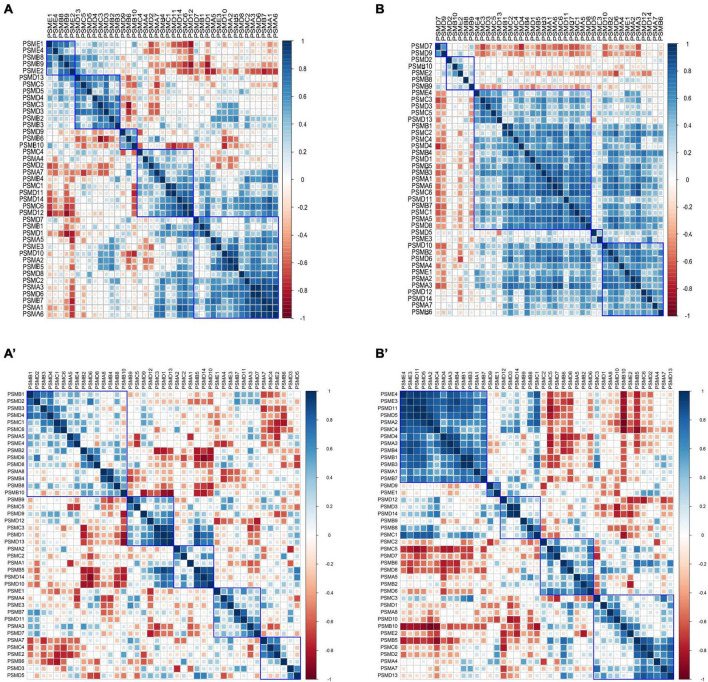
Heatmap of correlation coefficient matrices of proteasome subunits. **(A,A’)** Pearson correlation coefficient matrix between proteasome subunits in the control group, with a relatively uniform distribution. **(B,B’)** Pearson correlation coefficient between proteasome subunits in the AD group, with most subunits clustered into highly positively correlated clusters. The subplots **(A,B)** are from GSE1297, and subplots **(A’,B’)** are from GSE28146.

The Pearson correlation coefficients of intersubunit of the proteasome were calculated in 9 controls and 22 AD subjects of varying severity using the GSE1297 dataset. The heat maps of the correlation coefficient matrix were drawn, respectively (Figures 6A, B). Interestingly, we observed the distribution of correlation coefficients between proteasome subunits was relatively uniform in the control group (Figure 6A). In contrast, the overall correlation coefficient of the proteasome in the AD group was significantly enhanced. Half of the subunits were coaggregated to integrate highly positively correlated clusters (Figure 6B). In addition, we used GSE28146 as an independent validation (Figures 6A’, B’). It suggests that existing proteasomes become more active to counterbalance the reduction in the total number of proteasomes. This activity is manifested as increased correlation coefficients between subunits within each proteasome. That is, the coaggregation interactions of existing proteasome subunits became stronger, thus improving coordination. Therefore, the degradation efficiency of the existing proteasome became higher. This counteracted the abnormal accumulation of toxic proteins caused by decreased total proteasome number.

### 3.4 Candidate proteasome dysregulated subunits significantly correlation with AD

#### 3.4.1 Identification of AD-associated proteasomal genes by correlation coefficient *T*-test

**FIGURE 7:**
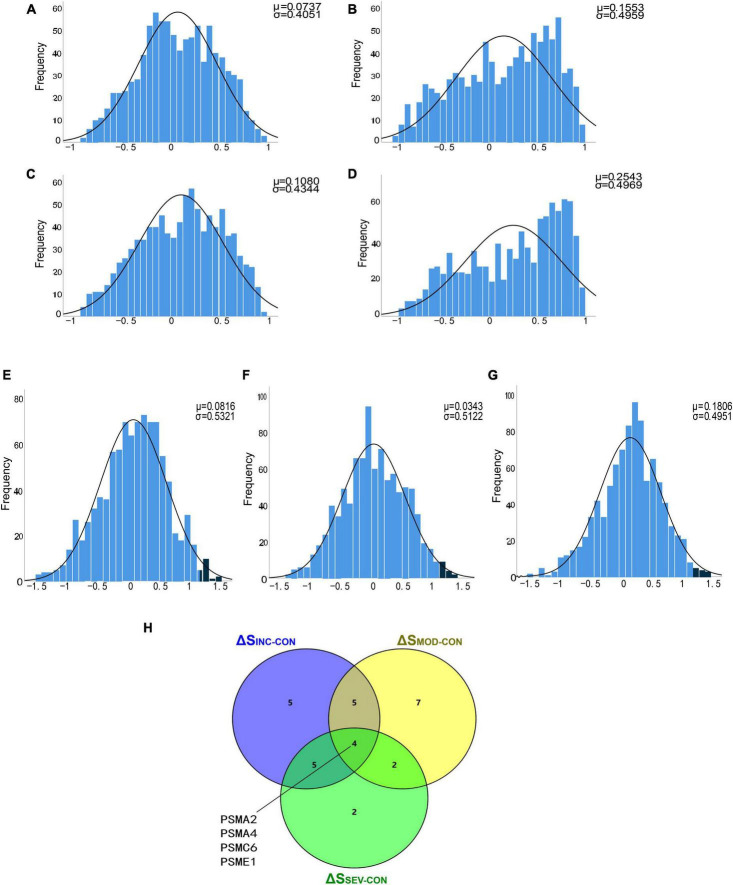
Identification of proteasomal hub subunits in AD. **(A–D)** The statistical histograms of the correlation coefficient matrix among proteasomal subunits in the four groups of “Control,” “Incipient AD,” “Moderate AD,” and “Severe AD,” respectively. The horizontal axis represents the magnitude of the correlation coefficient, ranging from −1 to 1, and the vertical axis represents the frequency of each interval element. The black curve corresponds to the normal distribution curve. The correlation coefficient distribution frequency can be observed to the right skewed from the three AD groups, indicating an increase in the number of positively correlated genes; **(E–G)** Histograms of the difference matrices statistics for the Incipient, Moderate, and Severe groups minus the Control group, respectively. The horizontal axis represents the magnitude of the difference in correlation coefficients, ranging from the minimum value to the maximum value. Three sets conform to the T-distribution. The dark blue areas on the right side indicate subsets of this set beyond 2 standard deviations from the mean, respectively; **(H)** Venn diagram showed the four crossover genes shared by the set Δ*S*_*INC*−*CON*_, Δ*S*_*MOD*−*CON*_ and Δ*S*_*SEV*−*CON*_.

To filter out significantly hyperactive proteasome genes, a *T*-test of correlation coefficients was utilized. Firstly, the correlation coefficient matrices of the whole proteasome subunits were calculated for the four groups in the GSE1297 dataset separately. Statistical histograms were then constructed based on these four multisets individually (Figures 7A–D). Statistically, we found that there were intergroup differences among the four groups. Compared to the Control, the correlation coefficients between genes in the AD groups were enriched toward a highly positive correlation. This again validated the enhanced coordination between proteasome subunits. Next, we counted the multiset of difference matrices of gene correlation coefficients, respectively. Three statistical histograms were constructed based on the minimum and maximum of the multiset (Figures 7E–G). All histograms approximately conform to the T-distribution.

**FIGURE 8:**
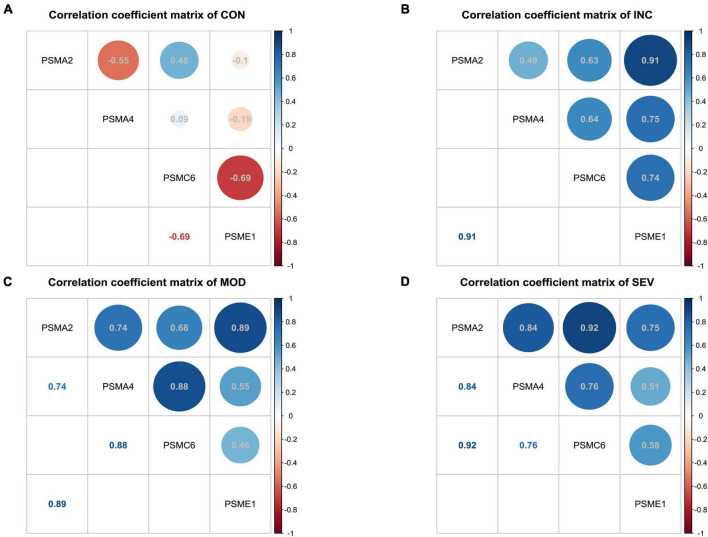
Variation of correlation of four hub genes in AD. **(A–D)** Heatmap of correlation coefficient matrices of four significant genes of proteasome in AD. From left to right, the matrix of correlation coefficients for the four genes in Control, Incipient, Moderate, and Severe, respectively.

To further discover the hub subunits, significant subsets of genes in each of the three difference matrices were extracted based on the three-sigma rule of thumb. As shown in the dark blue areas of Figures 7E–G. The difference correlation coefficients of the genes in these subsets all exceeded more than a twofold standard deviation (2σ). Then overlapped three subsets, the four crossover genes, PSMA2, PSMA4, PSMC6, and PSME1, were eventually identified (Figure 7H). The variation in the correlation coefficients of these four genes with disease progression was then analyzed (Figures 8A–D). These four genes showed statistically significant intercorrelation compared to the other genes.

#### 3.4.2 Identification of PSMC6 as a critical activator of proteasome in AD

**FIGURE 9:**
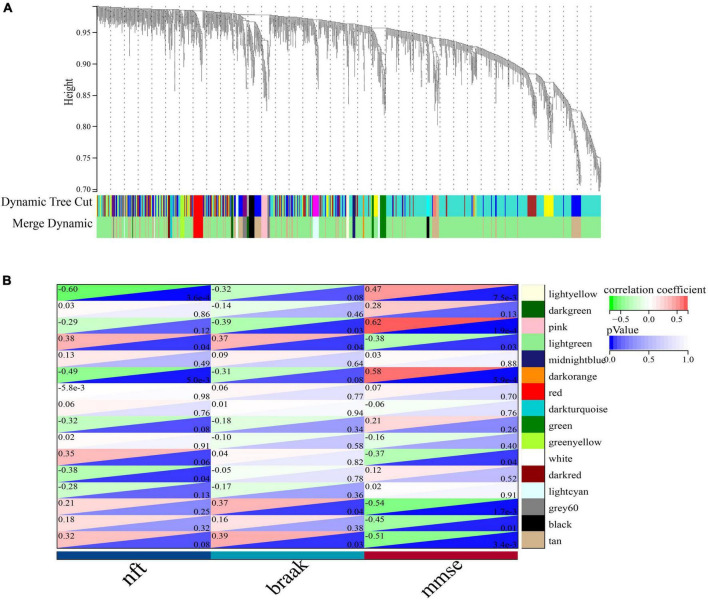
Co-expression network of significant genes in AD. **(A)** Clustering tree dendrogram of co-expression modules. Different colors represent similarity clustering at varying degrees. **(B)** Correlation analysis of light green modules with clinical status, each row represents a module and each column represents clinical status.

**FIGURE 10:**
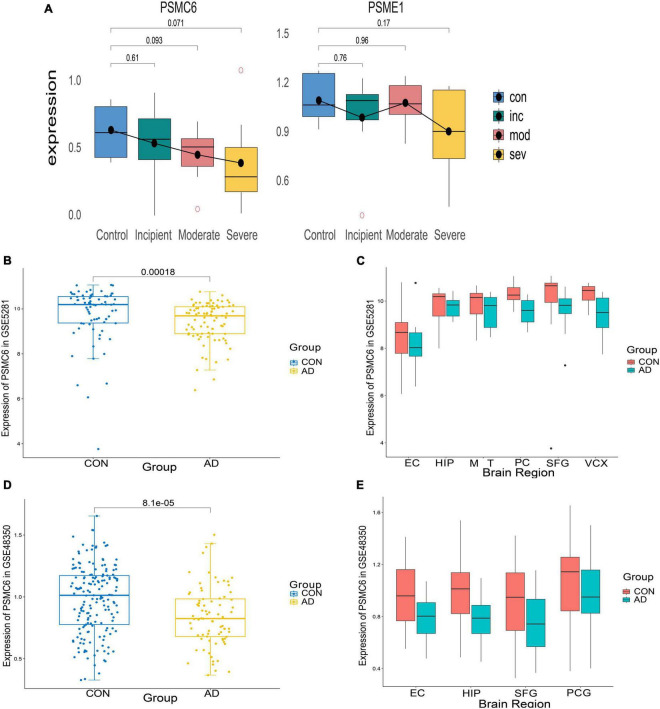
Expression of differential genes in AD patients and controls. **(A)** Boxplots of differential expression of PSMC6 and PSME1 according to the GSE1297 dataset. **(B,C)** From left to right, respectively depict the boxplots illustrating the overall expression differences of PSMC6 between AD and Control groups in the GSE5281 dataset, along with detailed expression variations across six distinct brain regions. These regions include the entorhinal cortex (EC), hippocampus (HIP), middle temporal gyrus (MTG), posterior cingulate (PC), superior frontal gyrus (SFG), and primary visual cortex (VCX). **(D,E)** Boxplots of the overall expression differences between AD and Control PSMC6 in GSE48350 with detailed descriptions of the expression changes in four different brain regions. Notably, PCG refers to the post-central gyrus.

To identify important activators, weighted gene co-expression network analysis (WGNCA) was used. The top 50% of genes of standard deviation of the GSE1297 gene expression profile were used as input and 16 gene expression modules were obtained (Figures 9A, B). Among them, only the light green module was significantly associated with the AD clinical features MMSE, NFT, and Braak. We then set the module membership (MM) threshold to 0.9 and the gene significance correlation (GS) threshold to 0.3. The results still contained PSMC6. This suggests that PSMC6 is not only significantly associated with other genes, but also strongly associated with clinical features. Therefore, we further explored the expression pattern of PSMC6 with AD by multiple datasets. The results showed that the expression of PSMC6 decreased monotonically with disease severity (Figures 10A, B, D). In addition, it revealed that PSMC6 was downregulated in all brain regions involved compared to the Control (Figures 10C, E). PSMC6, as the number of the ATP energy ring, provides energy for unfolding and straightening abnormal substrates on the one hand, and on the other hand, regulates the opening of the CP gate. Thus, a decrease in PSMC6 indicates a decrease in the efficiency of ATP-dependent 26S proteasomal degradation. Moreover, PSMC6 is more closely associated with AD than PSME1. This implies that activator 19S is more relevant to the pathologic of AD. So, we constructed a proteasome hub gene network with PSMA2, PSMA4, and PSMC6 as nodes.

### 3.5 The determinant and entropy of Hub gene network consistently declined with AD progression

**FIGURE 11:**
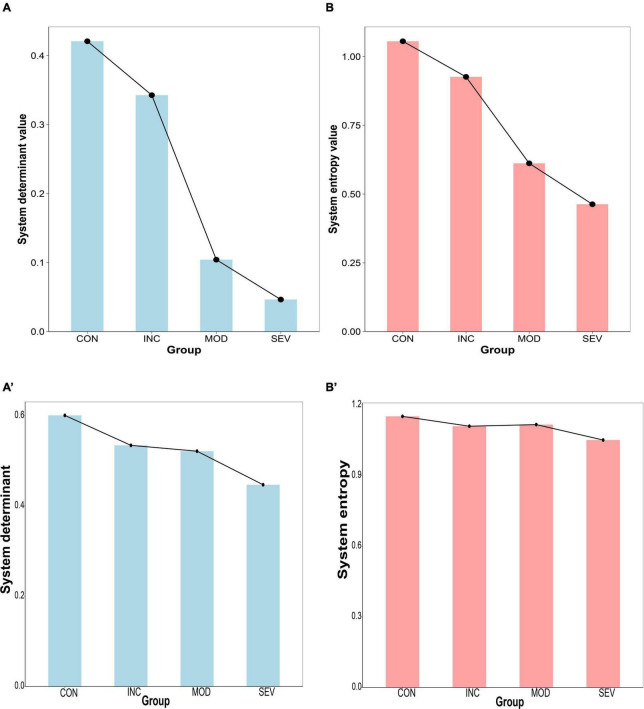
Histogram of system disturbance state of the hub gene network. (A,B) From left to right, the system determinant and entropy of the Pearson correlation coefficient matrices of PSMA2, PSMA4, and PSMC6 in GSE1297, respectively, and the results all continue to decrease with the AD process. **(A’,B’)** verification from GSE28146.

To characterize the relationship between the hub gene network and the pathological process of AD, determinant and entropy metrics were introduced. The determinant reflects the degree of correlation between dimensions. Entropy is used to measure the degree of disorder in a biological system. The expression of the hub gene network (PSMA2, PSMA4, PSMC6) of GSE1297 and GSE28146 were extracted. And constructed the Pearson correlation coefficient matrices of each of the four groups. Then the eigenvalues of the correlation coefficient matrix were obtained by matrix decomposition. And further calculated the determinants of the matrices and the systematic entropy and visualized them with a histogram (Figures 11A, B). The results showed that both network system determinant and system entropy decreased continuously and significantly. Increased proteotoxicity of AD exacerbates proteasome impairment. Consequently, the existing proteasomes enhance intersubunit coordination to offset the reduction in the total number of proteasomes. This enhancement is particularly reflected in the hub gene network. Specifically, there is enhanced intercorrelation among PSMA2, PSMA4, and PSMC6. Taken as a whole, the intercorrelation among these three genes can be quantified by the determinant of the system. Thus, as AD progresses, the system determinant of this hub gene network continuously decreases indicating a gradual increased global coordination of the proteasome. From another perspective, toxic proteins exert great influence on the hub genes of damaged proteasomes. This implies that genes of the hub network are less susceptible to external random perturbations. That is, these genes have less independence and degree of freedom. As a result, the systematic disorder degree of 26S proteasome with PSMA2, PSMA4, and PSMC6 as the core subunits was reduced. This also suggests that the proteasome has strong resistance to protein damage from AD, and makes feedback to improve degradation efficiency. This partly counteracted the deficiency of total proteasome, contributing to resisting proteotoxicity-induced cellular apoptosis. Therefore, the system entropy continued to decrease with the AD process.

### 3.6 The entropy of the determinant of the hub gene network consistently declined with AD progression

**FIGURE 12:**
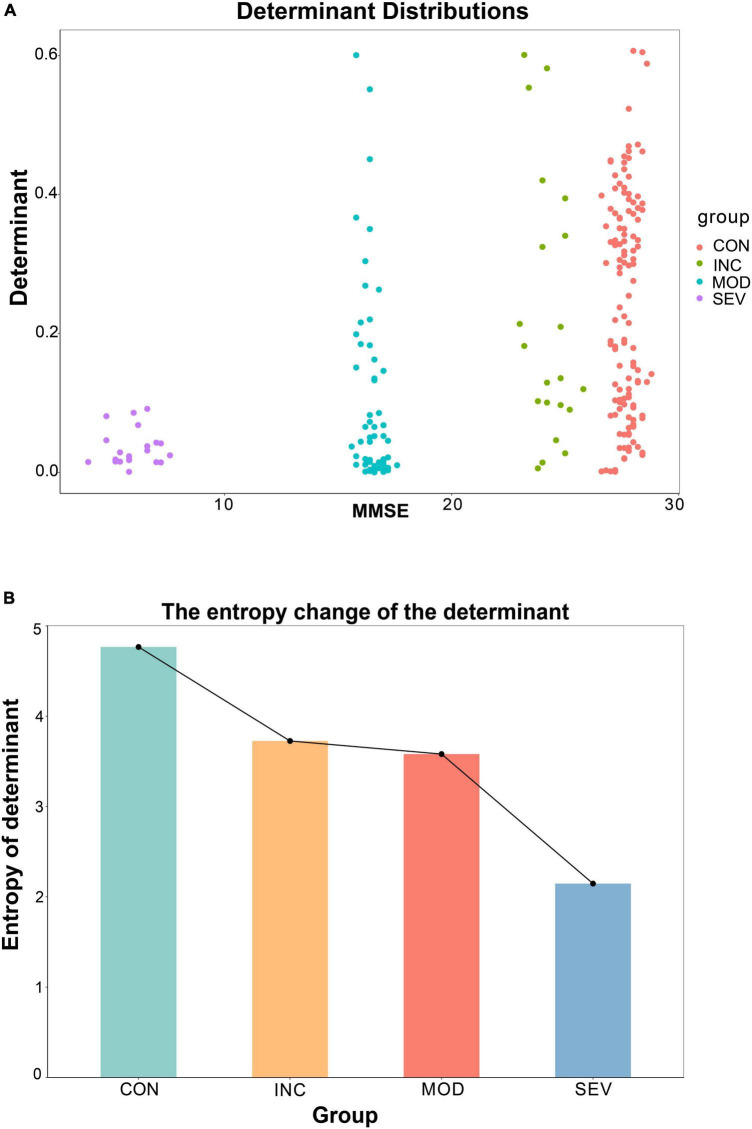
Variation of the entropy of the determinant of the hub gene network. **(A)** Relationship between the determinants of sample correlation coefficient matrices of the hub network and the average MMSE metrics for a specified sample *n* = 5. The larger the MMSE, the greater the randomness of the determinants, and the higher the scatter. **(B)** Entropy of each set of determinants for a specified sample of *n* = 5. The results showed a monotonically decreasing trend with increasing disease.

Additionally, the entropy of determinants was introduced to explore the consistency of the entropy of determinants of hub network molecules with the AD process. To avoid individual samples affecting the overall deviation, we sampled the same number of samples [denoted as *n* (*n* = 3,4,5)] from each group in GSE1297. Then constructed the correlation coefficient matrices based on the sample results for each group (“Control” = $C_{9}^{n}$, “Incipient AD” = $C_{7}^{n}$, “Moderate AD” = $C_{8}^{n}$ and “Severe AD”’ = $C_{7}^{n}$). And then statistically derived the determinants distributions and the information entropy of determinants for each group (Figures 12A, B). With the aggravation of AD, the MMSE, which measures cognitive ability, declined. Meanwhile, the value of the determinant transformed from being highly dispersed to being gradually clustered at the zero point (Figure 12A). The results are more significant and objective in data characterization with increasing sample size n (Supplementary Figure 2). In other words, the volume of the matrix gradually decreased, and the coordination of the three hub genes was getting stronger. This is consistent with previous results for the whole system determinant. Furthermore, we calculated the information entropy of the determinants for each group by using statistical histograms for each set. This study showed that the entropy of the determinants continued to decrease as the disease progressed (Figure 12B). This reveals that with the development of AD, the disorder degree of the determinant of the central gene network becomes weaker gradually. In other words, the diversity of determinants continues to decrease. Therefore, the 26S proteasome becomes more coordinated to focus on the degradation of abnormal proteins.

### 3.7 Composite candidate marker of PSMC6 and α-ring correlated with clinical indicator of AD

**FIGURE 13:**
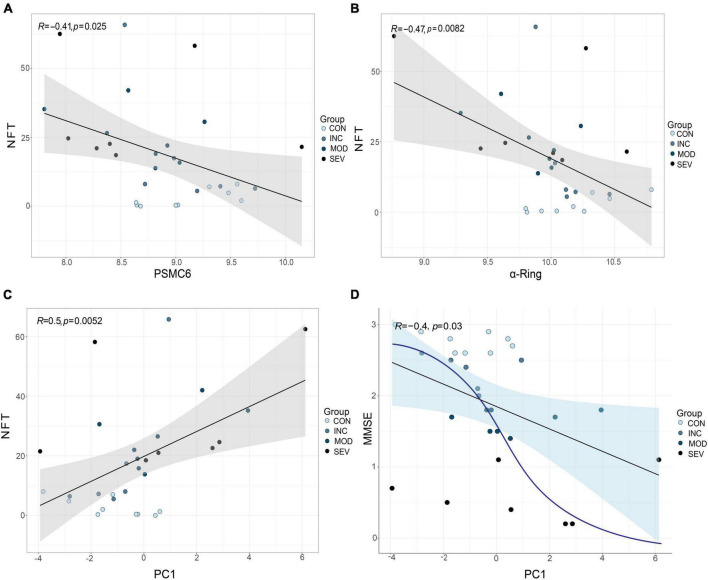
Validation of the role of RPT ring and α-ring in AD. **(A,B)** From left to right, are the linear fits between PSMC6 or α-ring, respectively, and the anatomical indicator NFT. **(C,D)** From left to right, linear correlations of PSMC6 and α-ring composite indexes with NFT and MMSE, respectively. R represents the correlation coefficient and *p*-value is the significance level.

The relationship between gene expression and clinical indicators was fitted by linear regression. Concretely, PSMC6 and the whole α-ring (PSMA1-PSMA7) of GSE1297 were extracted as a composite marker. An outlier sample due to a large postmortem interval (PMI) was excluded. Then we selected the first principal component PC1, which contained the largest feature, as a novel composite indicator. The results of the linear fit of PC1 and clinical indicators are shown in Figures 13A–D. The results showed that the correlation between PC1 and the clinical features is more significant than the individual with clinical without considering normal samples. Moreover, this correlation tends to be not linear as shown by the blue curve in the Figure 13D. The first principal component indicates the correlation between PSMC6 and α-ring. The higher the correlation, the greater the PC1 and the higher the severity of AD. In other words, clinically and pathologically, the first principal component values are available to preliminarily evaluate MMSE and NFT. This implies that, compared with a single gene, it is better to diagnose AD by extracting the hub proteasome genes PSMC6 and α-ring as quantifiable composite candidate markers.

### 3.8 The coherent effects on AD between PSMC6 and α-ring

**FIGURE 14:**
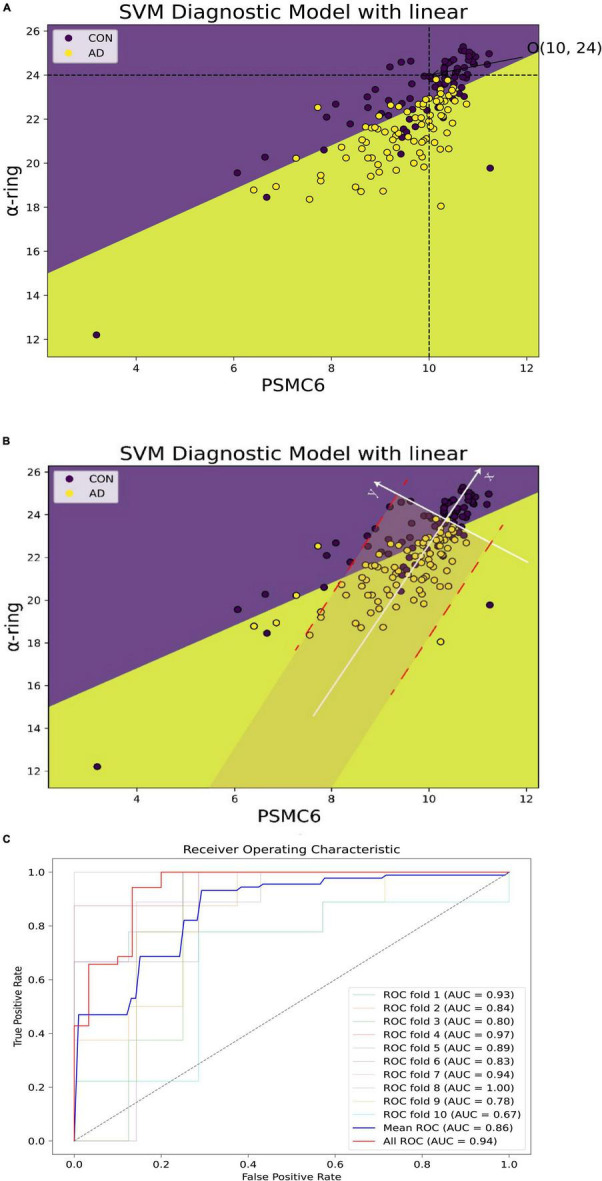
Alzheimer’s disease (AD) diagnostic models of PSMC6 and α-ring. **(A)** The horizontal coordinate is PSMC6 and the vertical coordinate is the average expression of α-ring. Patients with AD are shown in yellow and controls in purple. The point O (10, 24) formed by the intersection of the black dashed lines represented the threshold jointly determined by the two. There was little potential for AD to occur when the sample expression level was higher. **(B)** The new axis × formed by the white line represented the linear fit of the relationship between PSMC6 and α-ring for the AD samples remaining. It showed that the coherence between PSMC6 and α-ring is a candidate marker of AD. That is, if the expression levels of PSMC6 and α-ring are down-regulated coherently and the deviation between them is limited in a border (red line), the patient has a significant probability of AD risk. **(C)** Classification effect of the validation model. AUC indicates the area under the ROC curve, which could be used as the model classification prediction accuracy, when 0.5 < AUC < 1, the model classification effect is better and has the prediction value. The average AUC values for 10-fold cross-validation of the hub gene network are summarized, as well as the prediction accuracy of the microarray after principal component analysis based on the first and second principal components.

To further explore the synergistic roles of PSMC6 and α-ring in AD, the expression of PSMC6 and mean α-ring were extracted from GSE5281 as inputs to construct a support vector machine diagnostic model. As shown in Figure 14A, two characteristics were observed. Firstly, AD could be distinguished from normal brain samples quite intuitively with a hyperplane by the combination of PSMC6 and mean α-ring. Secondly, in AD progression, PSMC6 and α-ring synergize to form a threshold point (denoted as O). Individuals have little risk of developing AD when expression levels are above the threshold point. It suggested that the synergistic effect of PSMC6 and α-ring can be used to diagnose AD.

Based on coordinate transformation, we constructed a new coordinate system as in Figure 14B. Within this redefined coordinate framework, the *X*-axis is the linear regression trendline of PSMC6 and α-ring, while the *Y*-axis remains orthogonal to the newly defined *X*-axis. Some features found are as follows. Firstly, regarding the *X*-axis: 1. Diseased samples were predominantly concentrated in proximity to the *X*-axis. When mapped back to the original coordinate system, closer to the *X*-axis indicated a stronger correlation. This suggested that better coordination of the proteasome subunits is required to increase the efficiency of degrading aberrant proteins. Therefore, PSMC6 and α-ring were synergistically co-expressed at AD 2. The new coordinate system better captured the statistical relationship between disease probabilities. Notably, samples located to the left of the origin of the new *X*-axis were associated with a higher probability of disease, although a minor of control samples were also below this threshold. The expression of PSMC6 and α-ring decreased synergistically as the samples aggregated toward the negative half of the *X*-axis. Biologically, decreased expression of PSMC6 and α-ring indicated a reduction in the total number of proteasomes. AD originated from the accumulation of toxic substances, thereby exacerbating the condition. Secondly, concerning the features of the *Y*-axis: 1. Most cases were symmetrical around the positive and negative half-axis of the *Y*-axis. This pronounced symmetry reflected the correlation between PSMC6 and α-ring. It indicated a more coordinated interaction between hub genes pathologically. And these genes’ primary contributions were focused along the *X*-axis 2. The *Y*-axis also displayed the threshold characteristic. Data points of AD patients, denoted by yellow markers, clustered within a gray area defined by two parallel red dashed lines. This threshold indicated the deviation of the sample from the principal component, i.e., the degree of intergenic dysregulation. It signified that PSMC6 and α-ring were considered as a marker with the pathological progression, and their randomness and diversity are confined within specific thresholds.

The common characteristics of this new coordinate system underscored a pivotal observation: as AD advances, the accumulation of toxic proteins leads to aberrant proteasome subunit expression. It, in turn, prompted an elevated degradation efficiency of each proteasome. Based on these observed characteristics, the probability of AD increases when the *x*-axis decreases below a certain threshold and the *y*-axis is within a certain deviation threshold. On the one hand, outside the gray area, the probability of misclassifying a patient as a normal sample is about 5.75%. On the other hand, the probability of misclassifying a normal sample as AD is relatively higher within the gray area. In addition, to avoid coincidence, 10-fold cross-validation was applied and the respective ROC curves were plotted (Supplementary Figure 3), which ultimately summarized the predictive accuracy AUC values of the model that included all genes and with the hub network (Figure 14C).

## 4 Discussion

The aggregation of abnormal protein inclusions such as insoluble Aβ and tau are the major hallmarks of Alzheimer’s disease (AD) pathogenesis. These protein aggregates produce toxic substances that lead to neuronal degeneration (Wu et al., 2017). The proteasome is an important cellular regulator responsible for cellular quality control (Kim et al., 2018). It has been shown that proteasome subunits have profound effects on the solubility and aggregation of specific proteins in the developing brain (Kim et al., 2018; Rashid and Niklison-Chirou, 2019; Chocron et al., 2022). In AD, toxic protein aggregates damage intracellular proteostasis, forcing the proteasome to respond (Chondrogianni et al., 2015; Sahu et al., 2021). This dynamic relationship between proteasome function and toxic protein aggregation highlights the intricate interactions in AD pathology. By elucidating these complex molecules, we aim to deepen the understanding of AD pathogenesis and provide some help for potential therapeutic avenues.

In this study, we focused on the proteasome complex. Firstly, we analyzed the differences in proteasome expression between brain tissue from AD patients and Controls. Our observations showed that the expression of the vast majority of proteasome subunits was downregulated in brain tissue from AD patients compared to controls (refer to Figure 5). Of particular note, the expression of PSMC6 was significantly reduced and differentially expressed in different brain regions (see Figure 10). Subsequently, we performed a comparison of the correlation of all proteasome subunits between the two groups. Our study found that the intersubunit correlation coefficients of the entire proteasome were significantly enhanced and most of the subunits showed highly positive correlations (see Figure 6). Finally, through an in-depth analysis of the proteasomal hub gene network, we found that the determinant and entropy of the entire proteasome system continued to decrease as the disease progressed (refer to Figure 11).

Based on the above data characterization. We deduced that abnormal expression of proteasomal subunits is induced with toxicant infestation during the AD process, with the most affected being the α-ring and PSMC6. α-ring assembly is the initiation of proteasome synthesis and provides a structural template for the subsequent step-by-step assembly of the β-ring (Schnell et al., 2021, 2022). The protein encoded by PSMC6 is one of the 19S RPT rings of proteasome. It anchors the 19S to the 20S to form the 26S proteasome (Livneh et al., 2016; Dong et al., 2019). The reduction of these genes reveals a decrease in the total number of 26S proteasomes. This forces each of the existing effective proteasomes to enhance its activity and improve inter-subunit coordination for more efficient degradation of toxic aggregates. In addition, the disorder degree of the hub network of the whole proteasome is also reduced, to improve the anti-toxicity of the proteasome itself to counteract the reduction in its total number.

Based on the significant association of 26S proteasome with pathologic NFT. We further found that after excluding normal samples, the composite 26S proteasome marker, represented by the first principal component of C6 and α rings, was significantly negatively correlated with clinical MMSE. The stronger the marker correlation, the lower the degree of dementia. Therefore, in this paper, the synergistic effect of PSMC6 and α-ring composite candidate marker was utilized, then then SVM was applied to achieve the classification and prediction of AD. Referring to Figure 14, the synergistic downregulation of PSMC6 and α-ring leads to a significant increase in the potential risk of developing AD.

In this paper, *T*-tests, determinants, and entropy are innovatively introduced into the screening and validation of genes. Firstly, the most significant active subunit in the proteasome complex was screened out by a *T*-test of the difference matrix of the correlation coefficient. This was then used as a seed network to validate the relationship between proteasome and AD. In the validation process, we introduced determinants and entropy as mathematical and physical concepts. These two are named biological system determinant and system entropy, respectively. And given new concepts to further characterize the global coordination and system disorder of life system. This may be a new application for detecting AD disorder and measuring system characteristics.

In sum, the metagene of the RTP energy ring, PSMC6, and the α-ring assembled by the PSMA family play a crucial role in the advancement and progression of AD. Our studies indicate that under-expression of PSMC6 and α-ring stimulates the activity of existing proteasome and its resistance to toxic substances, to increase individual degradation efficacy. Nevertheless, it does not fully compensate for the lack of the total proteasome, and eventually, it still leads to inefficient degradation of the ubiquitin-26S-proteasome system for abnormal proteins, which results in neuronal damage.

## 5 Conclusion

The pathological process of Alzheimer’s disease involves the accumulation of abnormal proteins. As a hydrolytic nanomachine of cell regulation and waste management, the proteasome is the endpoint for the ubiquitin-proteasome system degradation, orchestrating the elimination of damaged or misfolded proteins. 20S proteasome is a barrel-like structure with a narrow pore that exhibits regulated gating. It has three types of regulatory caps, including 19S, 11S, and PA200. This is important for preserving proteostasis relevant to brain health and disease. In this study, the relationship between proteasome and AD was explored, and the following five characteristics were observed.

1. The gene expression levels of proteasome subunits are downregulated with AD progression (Figure 5). That is, the total number of 26S proteasomes is decreased, and there are not enough proteasomes for degradation.
2. The correlation between two subunits of proteasome is enhanced in the process of disease progression (Figure 6). This suggests that the subunits of existing active proteasomes cooperate more closely with AD progression. That is, the efficiency of degradation of individual active proteasomes is improved.
3. The determinant of the correlation matrix of subunits decreases continuously as the disease progresses (Subfigure 11A). Where the determinant measures the global coordination of the system consisting of subunits (Figure 2), the smaller the determinant, the more coordinated the system. It should be noted that determinant does not refer to the correlation between two genes, it refers to the coordination of the system as a whole. The enhanced global correlation suggests that the system of subunits of active proteasome works more efficiently to counterbalance the reduction of the total number of proteasomes.
4. The entropy of the system of subunit expression of proteasome decreases continuously as the disease progresses (Subfigure 11B). Proteotoxicity causes the chaos of subunits expression of proteasome and disrupts the degradation function of the proteasome, entropy measures the chaos degree. That is, entropy reflects the robustness of the system to counter the interference caused by proteotoxicity. The smaller the entropy, the smaller the chaos degree, and the stronger the robustness. That is, with AD progression, the existing active proteasome holds stronger robustness to counter the interference caused by proteotoxicity.
5. The coherence between PSMC6 and α-ring is a candidate marker of AD (Figure 14). If the expression levels of PSMC6 and α-ring are down-regulated coherently and the deviation between them is limited in a border, the patient has a significant probability of AD risk.

In summary, with AD progression, existing active proteasomes enhance degradation efficiency significantly by improving their coordination. Thereby compensating for the reduced degradation ability resulting from the decline in the total number of proteasomes, and then maintaining cellular homeostasis.

In this paper, the candidate markers were obtained using GEO’s public dataset of AD microarray data by applying the bioinformatics method of WGCNA and multivariate statistical analysis of Student’s *t*-test, Pearson’s correlation coefficient matrix, and *t*-test of correlation coefficient difference matrix, and the machine learning was developed by support vector machine model to further validate the finding. Additionally, the concept of entropy was used to detect the disorder of the proteasome system, it was discovered that entropy is down-regulated continually with AD progression against system chaos caused by AD. Another conception of matrix determinant was used to detect the global coordination of proteasome in this paper, it was discovered that the coordination is enhanced to maintain the efficiency of degradation. The features of entropy and determinant suggest that active proteasomes resist the attack caused by AD like defenders, on the one hand, to protect themselves (entropy reduces), and on the other hand, to fight the enemy (determinant reduces). The two conceptions enrich the tools of bioinformatics.

This study has shortcomings and potential limitations known to the author as follows:

1. There is no direct biological experiment to validate the hypothesis. The research team in this paper focuses on bioinformatics and does not have the ability to conduct biological experiments. On the other hand, no suitable collaborators for biological experiments have been encountered. To offset the lack of biological experiments, this paper verifies the hypothesis from multiple independent perspectives.

2. The study’s reliance on microarray data seems outdated, as RNAseq technology provides superior sensitivity and accuracy. This paper is a multi-view analysis of the data. Multi-view analysis requires datasets to satisfy many conditions at the same time, which makes it difficult to find datasets that meet the criteria. To eliminate the influence of noise, the data are logarithmized and only the exponentials of the data are compared, i.e., only the magnitudes are compared.

## Disclosure

GEO belongs to public databases. The patients involved in the database have obtained ethical approval. Users can download relevant data for free for research and publish relevant articles. Our study is based on open-source data, so there are no ethical issues or other conflicts of interest.

## Data availability statement

The datasets presented in this study can be found in online repositories. The names of the repository/repositories and accession number(s) can be found in the article/Supplementary material.

## Ethics statement

GEO belongs to public databases. The patients involved in the database have obtained ethical approval. Users can download relevant data for free for research and publish relevant articles. Our study is based on open-source data, so there are no ethical issues or other conflicts of interest.

## Author contributions

JX: Conceptualization, Data curation, Formal analysis, Investigation, Methodology, Project administration, Software, Supervision, Validation, Visualization, Writing – original draft, Writing – review and editing. XP: Writing – review and editing, Formal analysis. LY: Methodology, Writing – review and editing. XS: Methodology, Writing – review and editing. CP: Funding acquisition, Resources, Supervision, Writing – review and editing, Conceptualization, Methodology.

## Footnotes

1
https://www.ncbi.nlm.nih.gov/geo/

2
http://sangerbox.com/
