# Supplementary material for: The coherence between PSMC6 and α-ring in the 26S proteasome is associated with Alzheimer’s disease
Source: Front Mol Neurosci. 2024 Jan 31;16:1330853. doi: 10.3389/fnmol.2023.1330853 (PMC10864545; doi:10.3389/fnmol.2023.1330853)
Supplement: Supplementary file 1 [file Table_1.DOCX]

Supplementary Material

# Supplementary Figures and Tables

## Supplementary Figures


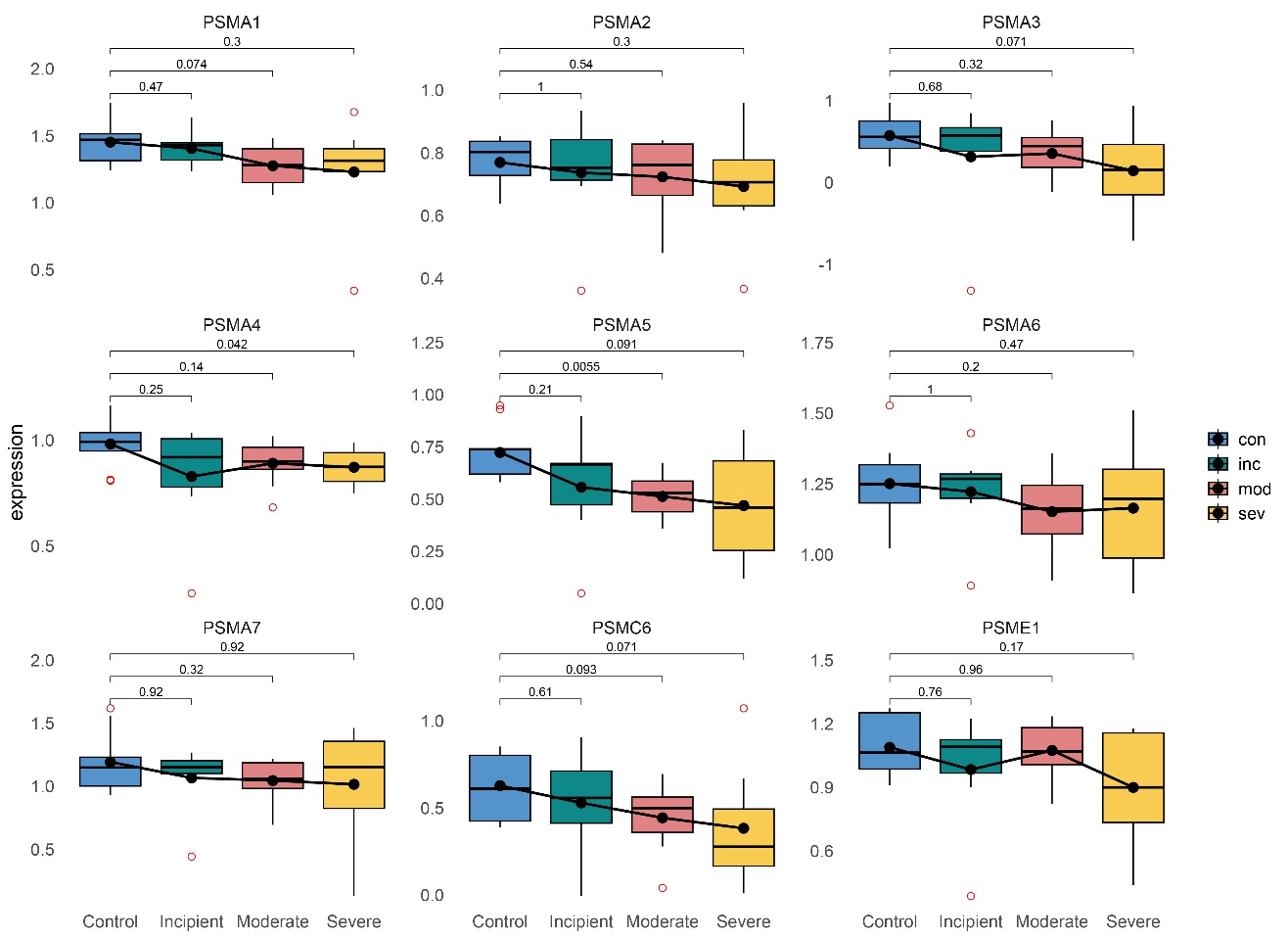


**Supplementary Figure 1.** Expression of hub genes. The figure shows in detail the expression of seven genes of the α ring (PSMA family), PSMC6, and PSME1 at four different severities in the GSE1297 dataset.


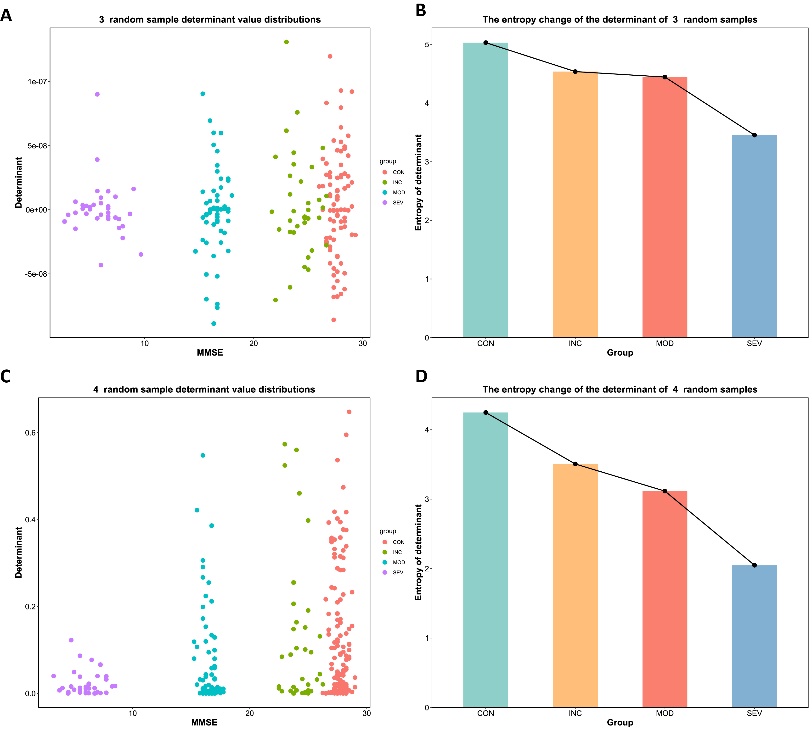


**Supplementary Figure 2.** Changes in the determinant entropy of the hub gene network with different sample sizes. (A-B) Sample capacity of 3; (C-D) Sample capacity of 4.


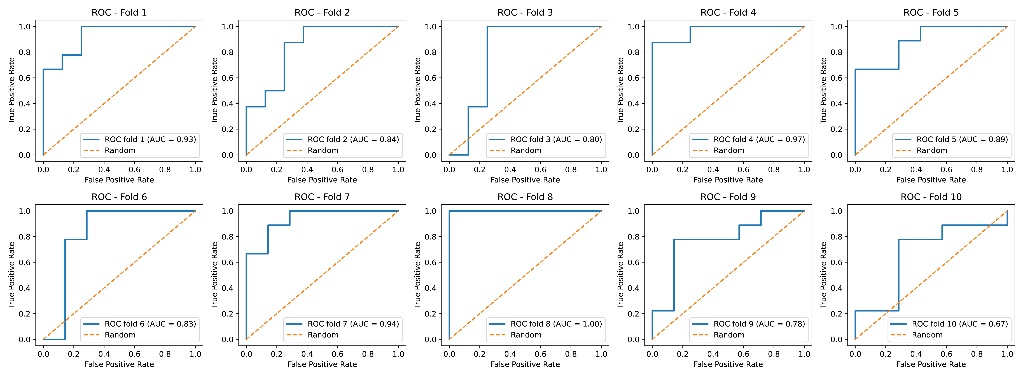


**Supplementary Figure 3.** Ten folds verify the classification specific effect of the model. AUC indicates the area under the ROC curve, which can be used as the prediction accuracy of model classification, when 0.5<AUC<1, the model classification effect is better and has prediction value.
